# Supplementary material for: ProTECT—Prediction of T-Cell Epitopes for Cancer Therapy
Source: Front Immunol. 2020 Nov 10;11:483296. doi: 10.3389/fimmu.2020.483296 (PMC7683782; doi:10.3389/fimmu.2020.483296)
Supplement: Supplementary file 1 [file DataSheet_1.docx]

***Supplementary Material***

# Supplementary Figures, Tables, and Files

## Supplementary Figures


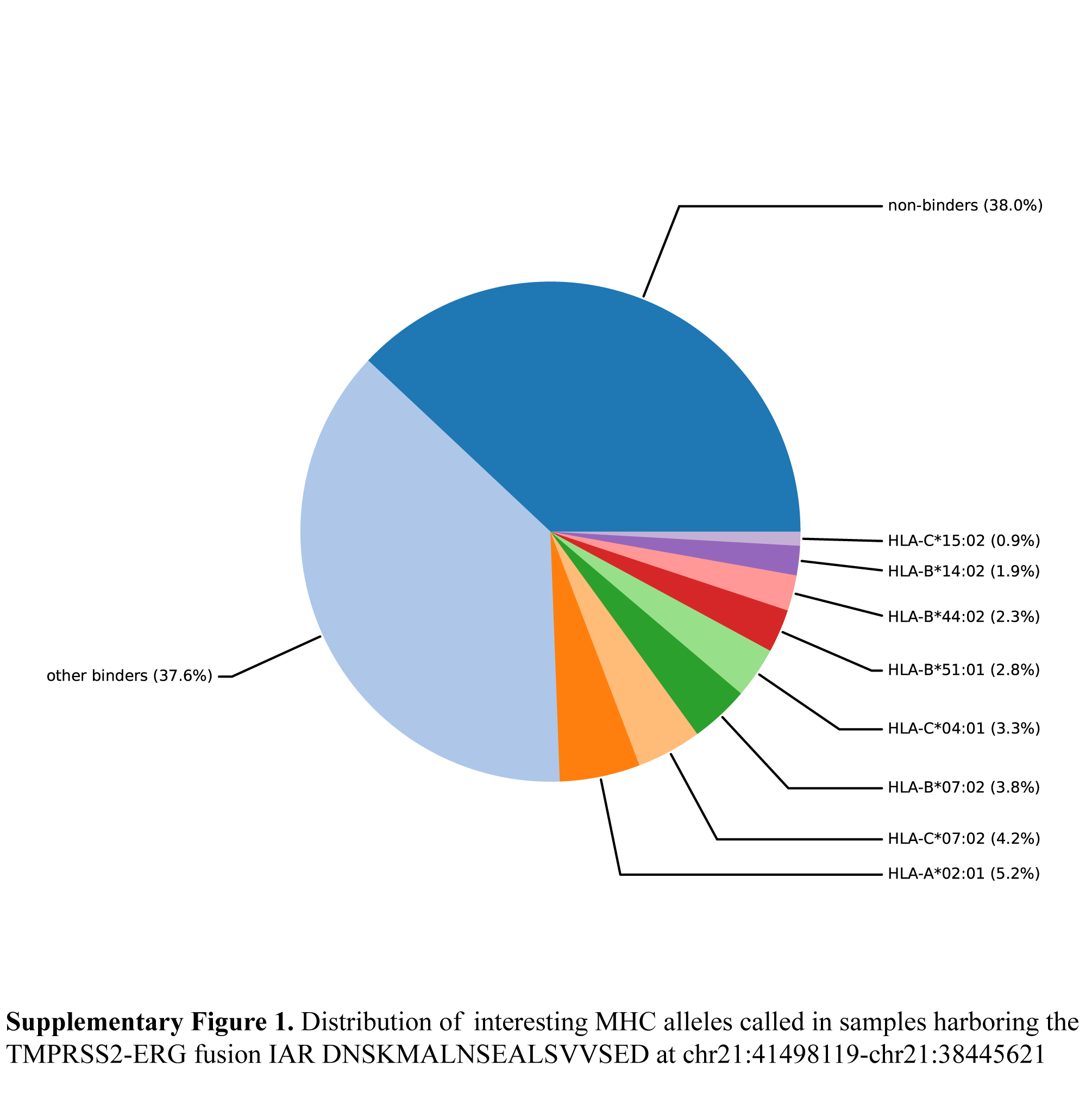


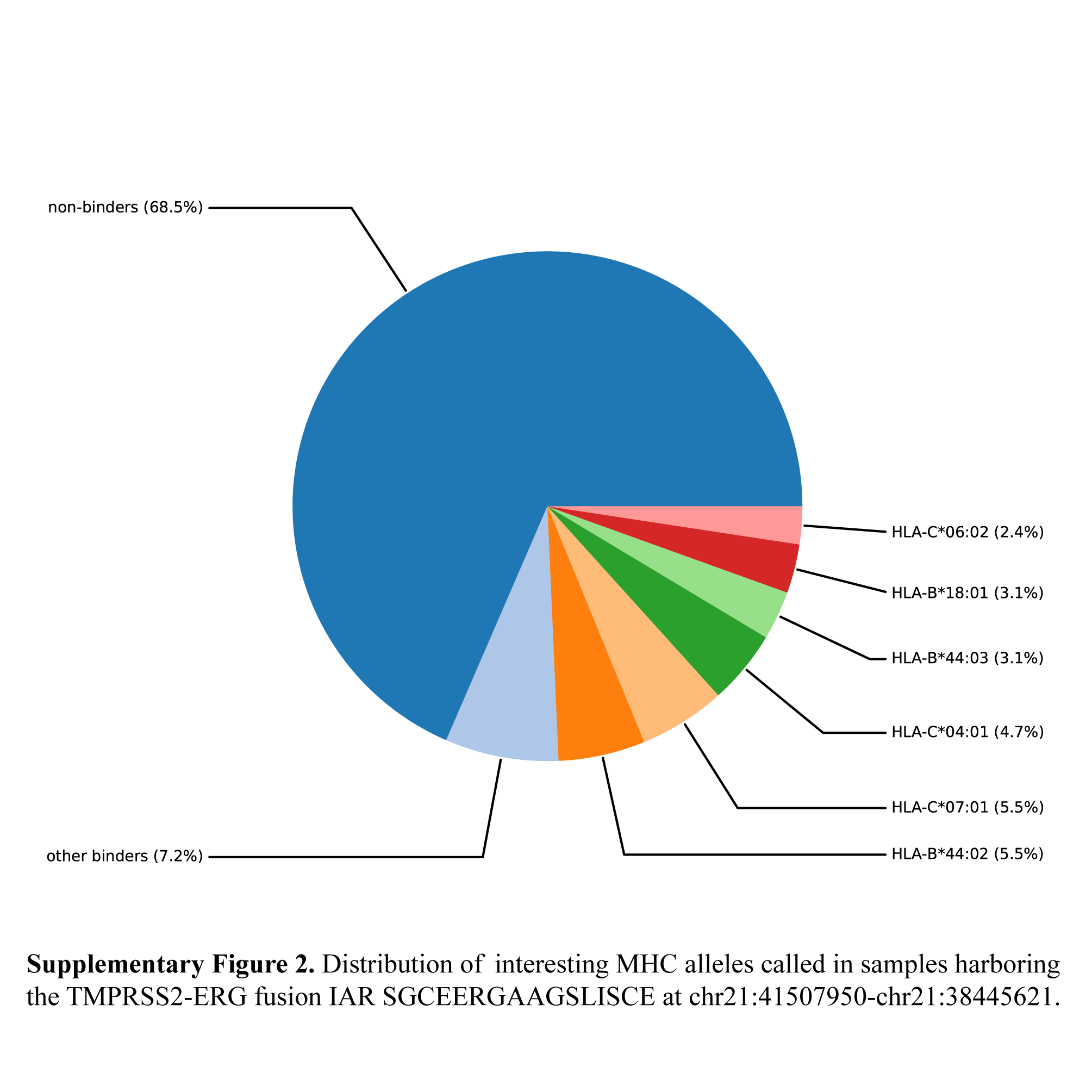


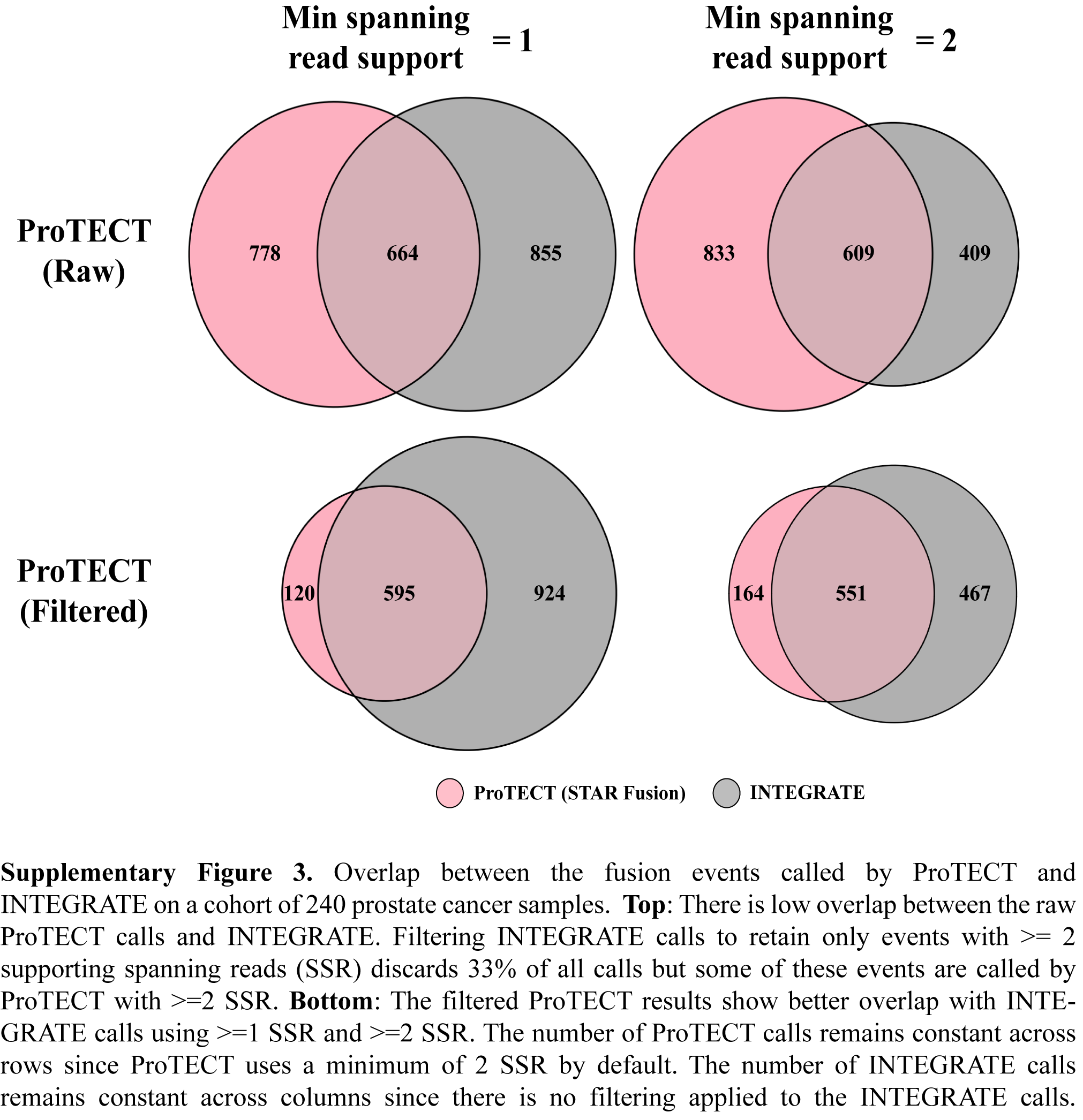


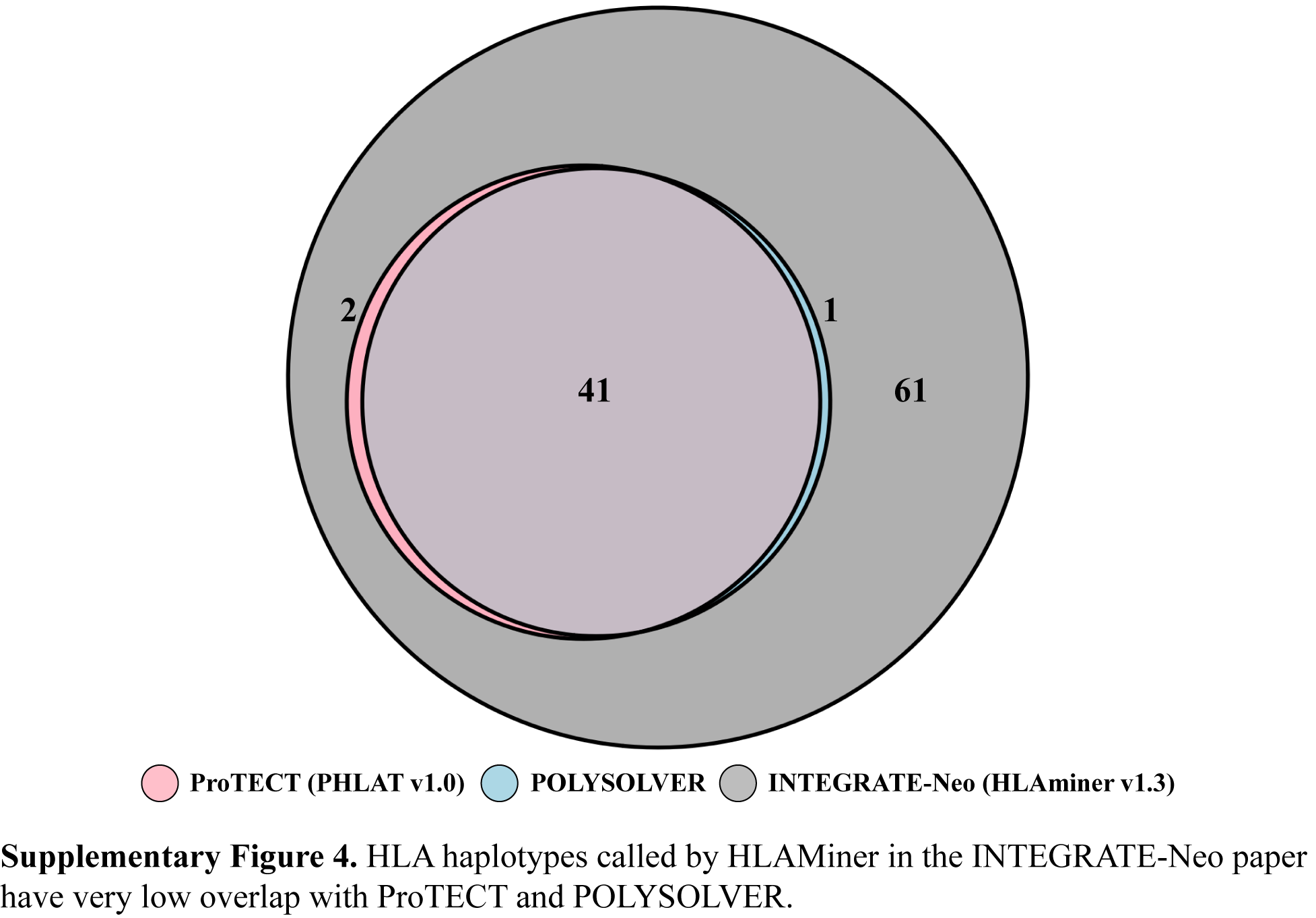


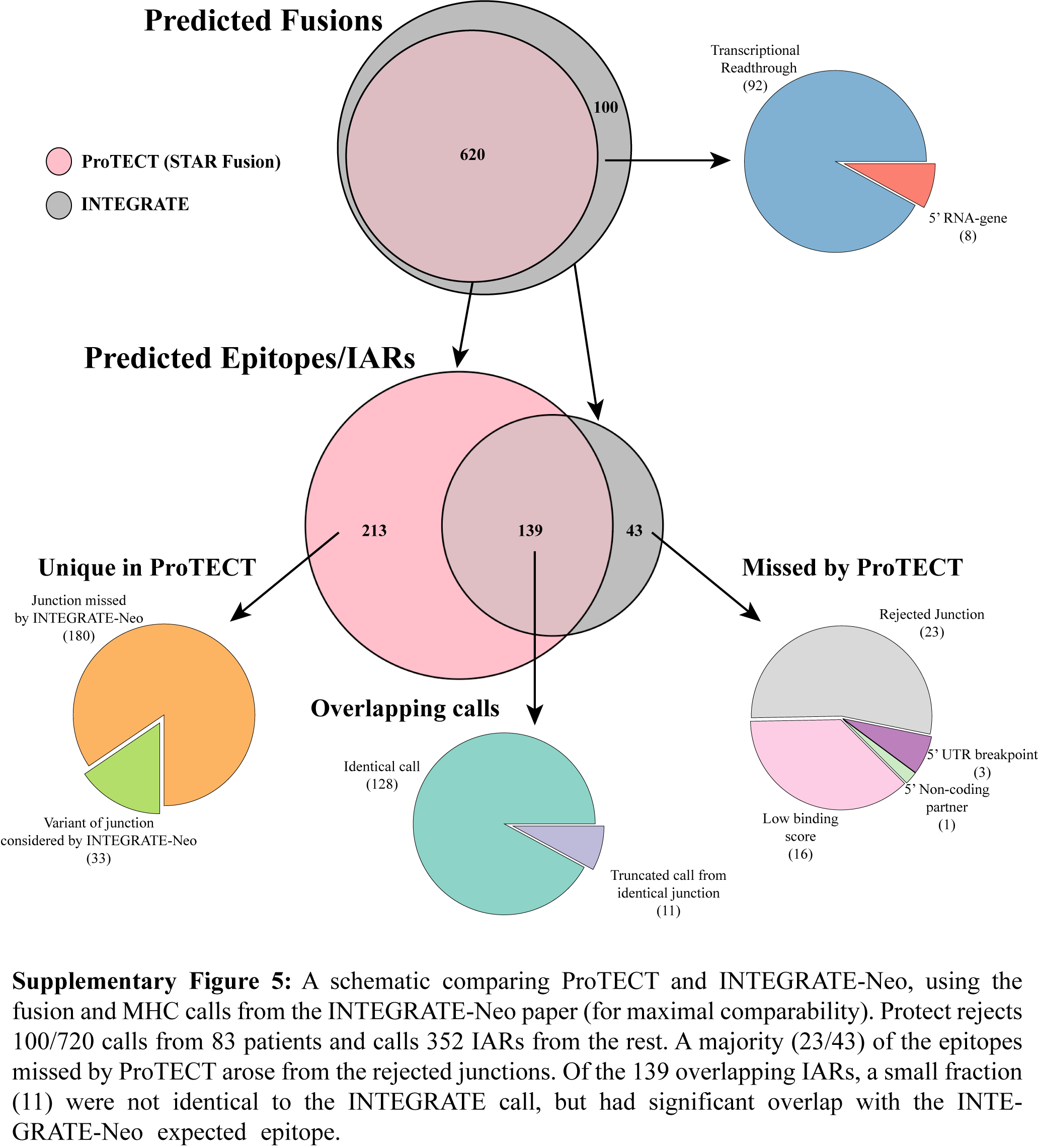


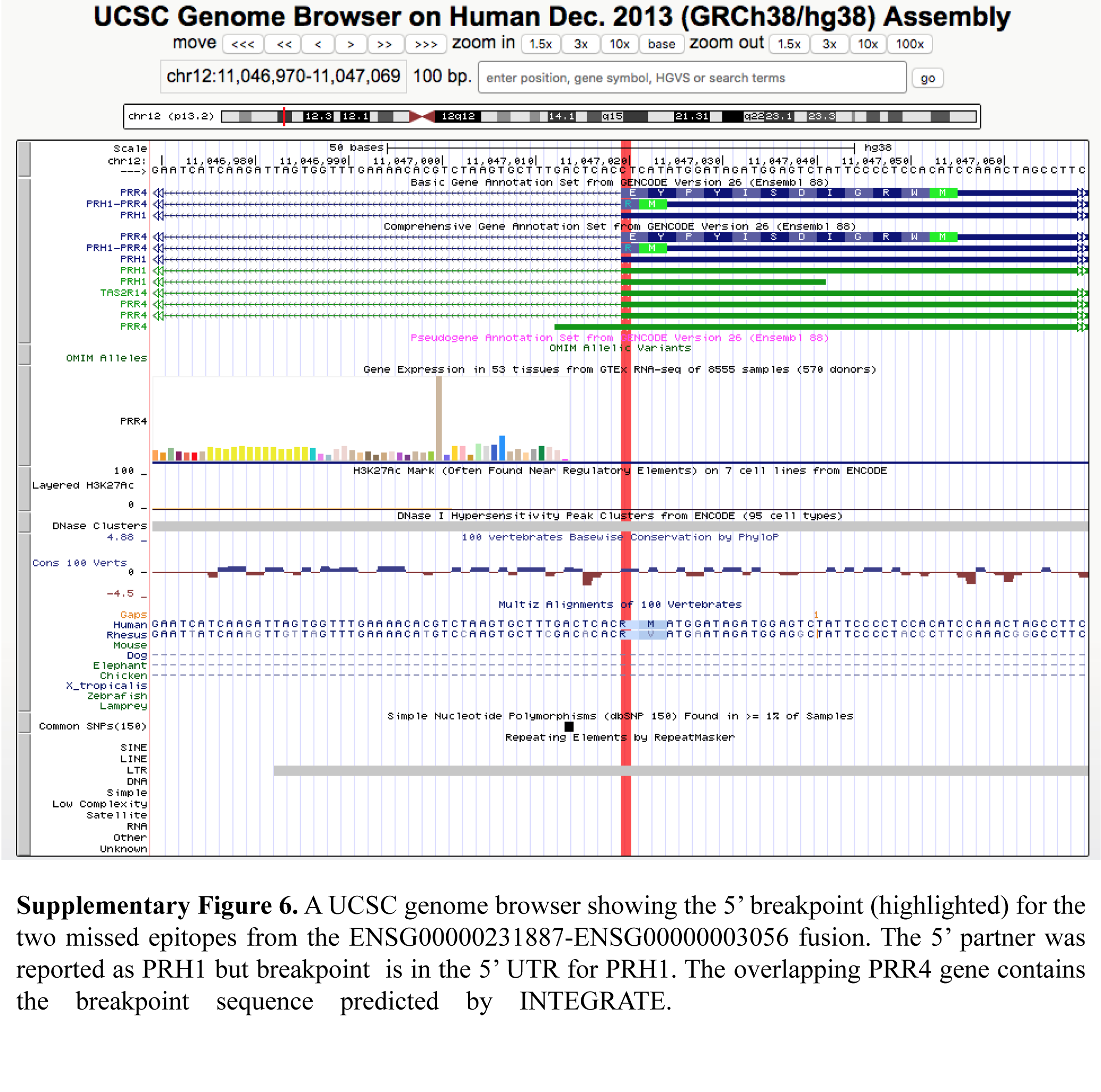


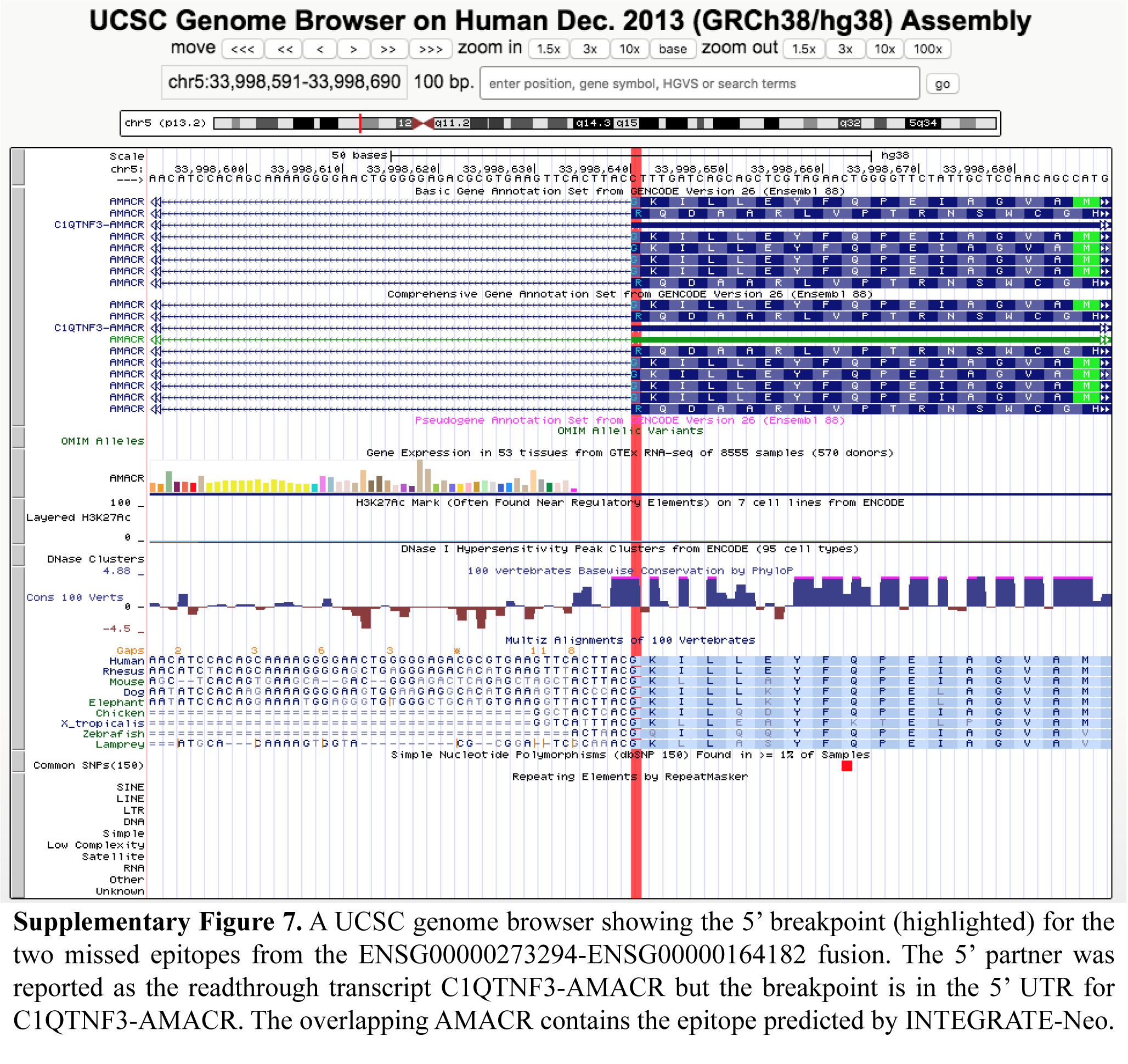


- 1. **Supplementary Tables**

**Supplementary Table 1.** The overall metrics for the 326 samples PRAD ProTECT run.This table includes all metrics captured for each file including input file sizes, and numbers of mutations called and accepted, peptides generated, and IARs predicted.

**Supplementary Table 2.** The ProTECT ranks for all 7 vaccine candidates tested by Carreno et.al. (1). Some variants were excluded from neoepitope prediction because the transcripts harboring those variants were in the bottom 10 percentile of expression for the sample. ^1^ARFGEF1:F1673L is the product of chr8:67204728G>T (ttC->ttA). However, we additionally identified the synonymous SNV chr8:67204729A>G (tTc->tCc) and found that every spanning RNASeq read in Mel21 Lymph Node, Skin (2012), and Skin (2013)+RNA1 harbored both events making the true codon change tTC->tCA, or F1673S. The IAR harboring this change was called in all samples. Skin (2013)+RNA2 had at least one read with only the first mutant hence F1673L was called in that patient. ^2^HSD17B7:H108Y in MEL38 is probably a miscall or an artifact of a different genome annotation as all transcripts of HSD17B7 in Gencode 29 have Leucine at position 108. The 2 uncalled variants in Mel218 were not identified as SNVs in our pipeline.

**Supplementary Table 3.** Ranked IARs predicted from all 8 samples described in Carreno et.al.(1).

**Supplementary Table 4.** Detailed reasons for rejecting INTEGRATE-Neo(2) predicted neoepitopes. Table A describes 4 breakpoints that were missed due to issues with the 5’ partner, Table B describes a breakpoint that should have failed similar to the case in Table 1, but were rescued by the prediction of a second, overlapping breakpoint, and Table C describes the INTEGRATE-Neo- and ProTECT-predicted binding affinity for the epitopes rejected as poor binders.

**2.3 Supplementary Files**

**Supplementary File 1:** A tarball containing all the results from running ProTECT on 326 samples in the TCGA PRAD cohort.

**Supplementary File 2:** A tarball containing all the results from running ProTECT on 8 Melanoma samples described by Carreno et.al. (1).

**Supplementary File 3:** A tarball containing all the results from running ProTECT on 83 samples from the TCGA PRAD cohort, described by Zhang et.al (2). Specifically, this contains the results from running ProTECT using the fusions and HLA calls described in Zhang et.al.

**3 references**

1. Carreno BM, Magrini V, Becker-Hapak M, Kaabinejadian S, Hundal J, Petti AA, Ly A, Lie W-R, Hildebrand WH, Mardis ER, et al. A dendritic cell vaccine increases the breadth and diversity of melanoma neoantigen-specific T cells. Science (2015). doi:10.1126/science.aaa3828

2. Zhang J, Mardis ER, Maher CA. INTEGRATE-neo: a pipeline for personalized gene fusion neoantigen discovery. Bioinformatics (2017) 33:555–557. doi:10.1093/bioinformatics/btw674
